# Tribological Behavior and Microstructural Analysis of Atmospheric Plasma Spray Deposited Thin Coatings on Cardan Cross Spindles

**DOI:** 10.3390/ma14237322

**Published:** 2021-11-30

**Authors:** Corneliu Munteanu, Viorel Paleu, Bogdan Istrate, Anişoara Dascălu, Cornelia Cîrlan Paleu, Shubrajit Bhaumik, Ana Diana Ancaş

**Affiliations:** 1 Mechanical Engineering, Mechatronics and Robotics Department, Mechanical Engineering Faculty, Gheorghe Asachi Technical University of Iaşi, 63 D. Mangeron Blvd., 700050 Iaşi, Romania; cornelmun@gmail.com (C.M.); bogdan_istrate1@yahoo.com (B.I.); naik_6219@yahoo.com (A.D.); 2 Technical Sciences Academy of Romania, 26 Dacia Blvd., 030167 Bucharest, Romania; 3 Wagner High Quality Lubricants, Chennai 603202, India; shubrajit.bhaumik@wagner-german-oil.com; 4 Building Services Department, Faculty of Civil Engineering and Building Services, Gheorghe Asachi Technical University of Iaşi, 67 D. Mangeron, 700050 Iași, Romania

**Keywords:** coatings, atmospheric plasma spray (APS), grease lubrication, cardan joint, WC Co, AMSLER machine

## Abstract

Cardan joints are used in transmissions between misaligned shafts, as in all-wheel-drive (AWD) cars and railway applications. Their functioning is accompanied by heavy cyclical loads, with the cardan cross spindles subjected to intensive abrasive wear and pitting. In this paper, a solution to the mentioned issue is proposed, thin anti-wear coatings of Metco 32 and Metco 72 metallic powders deposited by atmospheric plasma spray (APS) on cylindrical samples cut from spindles of two cardan crosses made of 40Cr10 and RUL2 steel. The morphological analysis of the coated surfaces was realized by scanning electron microscopy (SEM), and the elemental composition of the tested samples was elaborated by energy-dispersive X-ray spectroscopy (EDS). To investigate the wear resistance of the coated samples in dry and grease-lubricated conditions, tests at constant load and constant speed were carried out using an AMSLER tribometer. The results of greased tests proved that the expulsion of the lubricant from the tribological contact occurred no matter the combination of coated or uncoated samples. During grease-lubricated tests of ten minutes, the least coefficient of friction was measured for uncoated specimens with better surface finishing; but in dry friction tests, the lowest values of the mean friction coefficients were obtained for the Metco 72 coatings. The porous coatings may act as lubricant reservoirs in long-lasting tests, providing a solution to the expulsion phenomenon of the lubricant to the boundary outside the area of the larger-diameter roller.

## 1. Introduction

Cardan joints, also known as Hook’s joints, are parts of the drivelines equipping all-wheel-drive (AWD) vehicles. They are also employed in railway vehicles, marine propulsion systems, and other industrial applications [1,2]. The cardan is one of the most utilized joints, used anytime there is a need to transmit the power and the motion between misaligned shafts. The major problem of a cardan joint is that it converts the constant input speed into a periodically fluctuating one [3]. To ensure a relative steady angular velocity, cardan joints are coupled in pairs, composing the so-called double cardan joint. In AWD cars, this transmission is subjected to the maximum motor torque, high dynamic loadings [2,4], and high torsional and lateral vibrations [5,6]. There are various AWD driveline configurations, presenting permanently engaged cardan joints or engaged “on-demand”, as in off-road hikes. The conditions of off-road paths, usually with a lot of dust, subject cardan joints to intense abrasive wear, especially when the seals are broken and dust enters the greased tribological pairs of the cardan. In addition, cardan joints may suffer intensive wear due to severe running conditions: unfavorable lubrication kinematics, heavy loads, shocks, and abrasive contamination of their greased contacts [1,7].

Cardan joint failure can result in very costly consequences. In automotive applications, sudden interruption of power transmission may result in serious problems and failures in other components of the driveline. Vesali et al. [7] presented the problematics of the spindles of the cardan cross, which fail by pitting due to high contact stress with the needles of the roller bearings equipping the hinges between the cardan yokes and its spider (cross). They proposed some design solutions to alleviate the problem. Stamenić et al. [8] emphasized that the spindle of the cardan cross must carry unfavorable distribution of the linear load. Wang and Qi [9] determined the reaction forces in the rolling bearings of drive systems with double cardan joints, revealing the high variation in these forces over time. These multiple causes reflect some of the reasons for intensified wear and premature failure of cardan joints.

An original idea is to prevent or reduce abrasive wear by coating the parts of cardan joints with anti-wear materials. Thermal spray deposition permits the deposition of various coatings (ceramic, metallic, ceramic-metallic, and polymeric) with a thickness of the deposited layer in the range of nanometers to millimeters [10]. Atmospheric thermal spray (APS) is one of the most productive and versatile thermal methods, allowing the deposition of any type of powder at a high rate, good quality, and low costs. This process is characterized by the fact that the particles composing the spraying beam do not interact with each other, the flux of the melted powder directed to hit and coat the material target on imposed trajectory [11]. Thus, after 5 to 20 successive covering layers deposited on the substrate, a multi-layer coating results, its thickness depending on the number of successive passes of the torch over the target. Kowalski [12] reported results on the wear resistance of diamond-like carbon (DLC) coatings in press joint components. Following the results of fatigue tests in rotational bending conditions of the shaft and microscopic analysis of surfaces, wear products in the form of buildups, abrasions and pits were detected. Cimpoeșu et al. [13] presented results on the corrosion resistance of a thin hydroxyapatite (HA) layer, deposited through electrophoresis on the Ti_4_Al_4_Zr metallic substrate, concluding that the TiO_2_ and HA composed layer provided homogeneous and good corrosion-resistant coating. Bita et al. [14] reported results on the adhesion of bio-ceramic coatings, as HA and bioactive glass, on MgCa bio-implants.

WC–Co-based alloys have a similar composition to the Metco powders used for APS deposition in our work, being very hard and wear resistant. Pogrebnyak et al. [15] developed a coating, based on a mixture of powders, 30–70% of Ni Cr B Si Fe (PG-19N-01) and WC–Co (hard alloy), by high-speed plasma jet with detonation, and the plasma detonation method. The fusion of the coating by plasma jet increased the hardness and the wear resistance of the coating several folds over the simple PG-19N-01 coating. Fernandes et al. [16] studied the effect of composition on carbides formation in the WC–M (M = Fe/Ni/Cr) powders, uniaxially pressed at 190 MPa and treated for one hour at 1400 °C in a vacuum of 20 Pa, emphasizing formed phases, binder composition, and the carbon content. Ndumia et al. [17] realized a review on various deposition methods of Fe-based coatings, underlying that the optimization of the spraying parameters conducts to denser and harder coatings with better wear resistance, but concluded that more studies are required to understand the influence of different elements on the properties of Fe-based coatings at high temperatures. Boas et al. [18] reported results on plasma laser-sputtered WC/Co anti-wear coating and found that the low-power laser keeps the substrate melting to a low degree, increasing the abrasive wear resistance of the coating. Guilemany et al. [19] studied the effect of debris formed by WC–Co coatings in contact with different counterparts (Al_2_O_3_, steel, Si_3_N_4_, etc.), showing that the main wear mechanism of WC–Co coatings is direct abrasion of joint surfaces (two-body abrasive wear) and abrasion due to wear debris (three-body abrasive wear), followed by carbide pull out. Bolledu et al. [20] performed a comparative study of nanostructured WC-17 wt.% Co coatings deposited by HVOF and APS with nitrogen and highlighted the better wear resistance of HVOF coatings. Nanoindentation tests on WC–Co coatings deposited by APS using H_2_ and He as plasma gases proved that decarburization strengthened the coating, and there was a correlation between macroscopic and nanomechanical behavior of the coatings [21]. Rahbar-kelishami et al. [22] examined the effect of the friction stirring process on the wear of thermally sputtered WC-12% Co coatings, finding that friction stirring increased hardness and wear resistance. Simunovic et al. [23] examined the process of patterning and optimization of thermally sputtered Ni-based self-fluxing alloy coatings, these coatings being close in chemical composition to the soft phase of the Metco 32 coating.

Khanna et al. [24] reported that PM 20 alloy (chromium carbide in Ni-Cr powder), WC/Co in Ni-Cr powder were deposited by APS and laser cladding to form hard and friction-resistant coatings, finding optimal coating hardness for 15–35% WC/Co in Ni-25Cr matrix, and arguing for the superiority of laser-cladded coating over APS. Kazamer et al. [25] studied the effect of blending WC–Co with Mo, obtaining the 80WC-16Co 4Mo powder. The results on microstructure, mechanical and tribological performances of this APS-deposited coating on C67 base steel proved that the new coating is less porous and harder, diminishing by 10% the wear rate in comparison with the WC–Co coating.

Bolelli et al. [26] reported that the resistance to abrasive wear of HVOF made Fe–Cr–Ni–Si–B–C (Colferoloy) coatings is less than that of Ni-based alloys and electroplated chromium. Instead, the addition of 20 wt.% and 40 wt.% of a WC–12 wt.% Co powder in Fe–Cr–Ni–Si–B–C composite coatings, deposited by HVOF and high-velocity air fuel (HVAF), mitigated the abrasive wear rate of Fe-based coatings by one order of magnitude [27].

Recent research conducted by Paleu et al. [28] proved that the APS-deposited coatings of AMDRY 1371 (Mo–NiCrFeBSiC) possess a wear intensity of the same order as Colferoloy–NiCrFeSiBC high-velocity oxygen fuel (HVOF)-deposited coating [26], provided that the thickness of the coating is optimized. Similar results were reported by Paleu et al. [29] for APS-deposited Al_2_O_3_ 40TiO_2_ (AMDRY 6250) coating.

After a thorough survey of the existing literature, we observed that there is no systematic published research regarding the microstructure and tribological behavior of the coatings deposited on cardan joint parts [30], the outcomes on grease lubrication conditions being a novelty. The current paper presents comparative results on the microstructure and tribological behavior of Metco 32 and Metco 72 coatings, deposited by the APS method on substrates obtained from two cardan spindles.

## 2. Materials and Methods

### 2.1. Materials

To obtain wear-resistant coatings, two powders were selected from the Oerlikon -Metco catalogue: Metco 32 and Metco 72. Metco 32 contains a hard phase of 80 wt.%; most of it is composed of tungsten carbide. The high concentration of WC confers to the coating extremely wear resistance, but a porous structure after spraying. The chemical symbol of Metco 32 is 20(Ni 17.5Cr 4Fe 4Si 4B 0.5C) 80(WC 12Co). Indicated in wt.%, in the hard phase of Metco 32, there is 88% WC and 12% Co. The matrix of 20% is made of 70% Ni, 17.5% Cr, 4% Fe, 4% B, 4% Si, and 0.5% C. The morphology of the Metco 32 powder particles is spheroidal, with nominal particle size distribution of −125 + 45 µm.

Metco 72 powder is 88WC 12Co, the chemical composition indicated by the catalogue as 81% W (min.), 11.5–13.0% Co, 5.25% C (max.), 1.5% Fe (max.), and other elements maximum 1.0%. Metco 72 particles are angular (blocky), with a nominal range −45 + 11 µm. As indicated by the manufacturer, Metco 72 produces very dense and well-bonded coatings, with excellent abrasive wear resistance at lower service temperatures (up to 500 °C).

**Figure 1:**
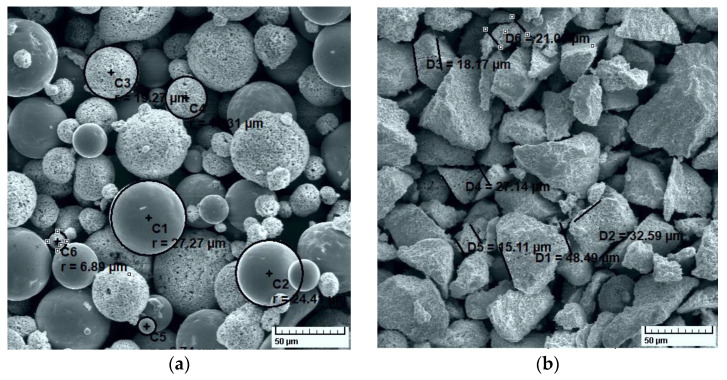
SEM images of Metco 32 (**a**), and Metco 72 (**b**), 500×.

SEM images for the used powders are shown in Figure 1, along with some measured particle sizes. The SEM images show that the Metco 32 particles are spheroidal, with sizes in the range of 6 µm to 28 µm. Due to the spherical shape, the powder provides improved flowability during spraying and gives the coatings better resistance to abrasion, fretting and erosion. The manufacturer recommends such coatings for wear protection. APS, Combustion Powder Thermospray (CPT) and HVOF are recommended as deposition processes of coatings for automotive, turbomachinery, petrochemical, agricultural and hydroelectric applications.

Metco 72 particles are irregular in shape, obtained by sintering and crushing, with measured sizes ranging from a few microns to a maximum of 50 μm. The material contains fine-grained carbides, also providing abrasion, erosion and fretting wear resistance, but only in dry, non-corrosive environments, our subsequent dry friction results proving this aspect.

The base material was obtained by cutting the spindles of cardan crosses equipping Mercedes Benz transporters and ARO Romanian AWD vehicles, denoted hereafter as Cardan 1 and Cardan 2, respectively. Cardan 1 is made of 40Cr10 steel (SR EN ISO 683-17 standard), containing 98.26% Fe, 0.39% C, 0.21% Si, 0.62% Mn, 0.03% P, 0.04% S, and 0.45% Cr. Cardan 2 is made of 100CrMnSi6-4 (according to SR EN ISO 683-17) or RUL2 steel (according to Romanian standards), with 96.1% Fe, 0.98% C, 0.42% Si, 1.03% Mn, 0.03% P, 0.02% S, and 1.42% Cr.

Substrates for deposition were prepared as follows: samples were cut to size, sandblasted with casting sand, cleaned of ethyl alcohol and positioned on a support plate for the deposition of coatings.

The obtained testing samples by combining the different cardan steels as base materials and the Metco 32 and 72 coatings are figured in Table 1.

The employed grease is Mobilux EP2, recommended for antifriction applications (rolling bearings, sleeve bearings, needle roller bearings), meeting the requirements of standard DIN 51825:2004-06-KP 2 K-20. The grease grade is NLGI2, and the thickener type is lithium. The base oil viscosity of the grease @ 40 °C is 160 mm^2^/s; penetration, 60×, 0.1 mm, ASTM D217; the Timken OK Load, according to ASTM D2509 is 40 kg; Four-Ball Extreme Pressure Test, Weld Point, kgf, ASTM D2596, is 250 Kgf; and Four-Ball Wear Test, Scar Diameter, mm, ASTM D2266, is 0.4 mm.

### 2.2. Methods

#### 2.2.1. Coating Deposition

The deposition of the coatings by the APS method was realized by Sulzer Metco 9MCE plasma jet equipment, made Sulzer & Metco, Pfäffikon, Switzerland. The process parameters are: spraying distance, 150 mm; current, 500 A; electric voltage, 630 V; argon flow, 50 m^3^/h; and hydrogen flow, 70 m^3^/h. For each coating, five successive passes of the deposition gun were accomplished.

#### 2.2.2. Microhardness Measurements and Scratch Tests

The Rockwell microhardness and the scratch tests of each coating carried out by using the equipment of the CETR UMT-2 micro-tribometer (Luleå, Sweden). Five similar tests were performed for each coating, and a mean value was obtained. The Rockwell diamond indenter has an opening angle of 120° ± 0.35°, radius 200 ± 10 μm, deviation from profile ±2 μm. The micro-indentation procedure consisted of a progressive increase in the indentation force from 0 to 5 N and back to nil value. The micro scratch tests of the coatings were realized at a constant force of 5 N and constant speed of 10 mm/min. The software equipping the tribometer is the CETR-UMT Test Viewer.

#### 2.2.3. Morphological and Structural Analyses

Scanning electron microscopy (SEM) equipment, model Quanta 200 3D Dual Beam (Waltham, MA, USA) was used to get images of the surface and cross-section of the coatings and substrates. The chemical characterization was made by energy-dispersive X-ray spectroscopy elemental mapping (EDS), model XFlash (Bruker, Billerica, MA, USA), [31].

For the cross-section SEM analysis, the coated specimens were cut and embedded in a BAK-R-type resin produced by Metkon. The samples were ground using seven sand papers of 180 to 1200 grit. The metallographic attack is performed by immersing the surface of the sample to be analyzed in a chemical reagent (Nital reagent, having a concentration of 5% of HNO_3_), each phase and constituent reacting differently; therefore, under the microscope, it will be possible to distinguish their shape and distribution as well as their nature.

#### 2.2.4. Surface Topography Measurement

A stylus profilometer model Form Talysurf 50 (made by Taylor Hobson, Leicester, UK), and the μltra Intra Form Talysurf software were employed for roughness measurements. Each measurement on the circumferential direction of the cylindrical samples was repeated three times, and mean values are reported.

#### 2.2.5. Friction and Wear Tests

The AMSLER machine is a bi-disc tribometer, type A135, made by Schaffhausen, Switzerland, the same as fabricated by Alfred J. AMSLER & Co., allowing friction and wear tests for various tribological pair configurations [32].

**Figure 2:**
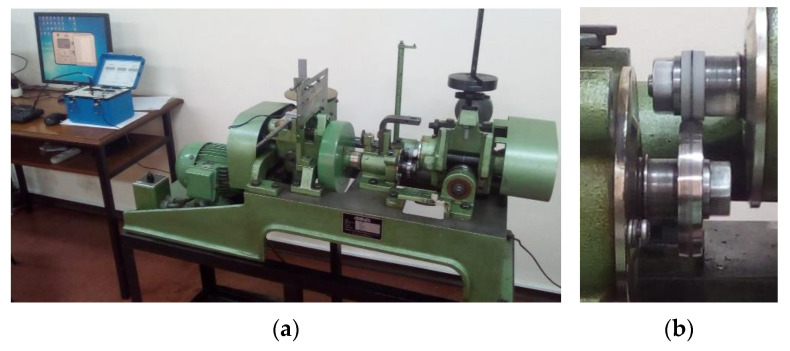
General view of the AMSLER machine (**a**), and bi-disc testing arrangement (**b**).

**Figure 3:**
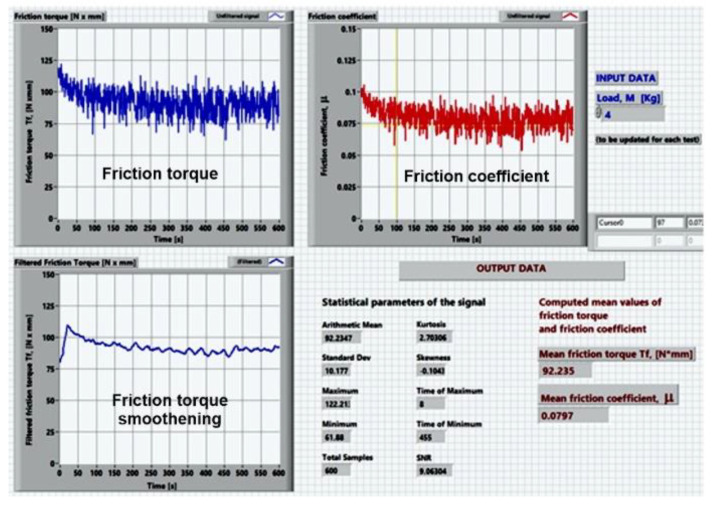
The LabVIEW interface for data post-processing.

The cylindrical test specimen, 30 mm in diameter and 10 mm thick, was mounted in the upper position and the lower rotating disc was made of AISI 52,100 bearing steel (hardness 60–64 HRC) with equal radii of 29.5 mm in both circumferential and axial directions and a thickness of 10 mm. The kinematic chains of the AMSLER machine provide a creepage (sliding to rolling ratio) of approximately 10% for equal diameters of the rollers, the ratio of the speeds realized by the upper and lower rollers being 0.906. In our case, the diameter of the upper disc is 30 mm, and the diameter of the lower disc is 59 mm, resulting in a sliding-to-rolling ratio of approximately 74%. The testing machine is fully described in our previous published papers [33,34,35], including the kinematic drivelines, data acquisition system, data acquisition chain calibration, and description of the main parts. A general view of the AMSLER machine is provided in Figure 2a. Figure 2b presents the tribological arrangement, including the upper coated testing specimen and the lower rolling bearing steel disk. To assure good repeatability, each test was repeated thrice, for both dry and grease-lubricated tests. According to Table 1, a total number of 36 friction tests were carried out for dry and grease-lubricated conditions, that is 18 dry tests and 18 lubricated tests. Mean values of the friction torque and coefficients of friction (CoF) were reported, besides the corresponding confidence limits. All the tests were carried out at a constant speed of 100 rpm, for 600 s each one. For dry friction, a constant load of 20 N was applied by the dead weights method. To obtain observable wear traces in rapid tests, the grease-lubricated tests were realized at a twofold constant load of 40 N. The friction torque was monitored by strain gauges measurements with the aid of a Vishay P3 tensometric bridge. To compute the mean friction torque and mean friction coefficient, and also to apply smoothening filtering to the acquired data and to estimate the statistical parameters (arithmetic mean, standard deviation, and signal to noise ratios), the acquired data were post-processed by the mean of a virtual instrument realized in LabVIEW 7.1 software, made National Instruments [33]. Figure 3 presents an image of the developed LabVIEW interface.

## 3. Results and Discussions

### 3.1. Hardness and Elasticity Modulus

**Figure 4:**
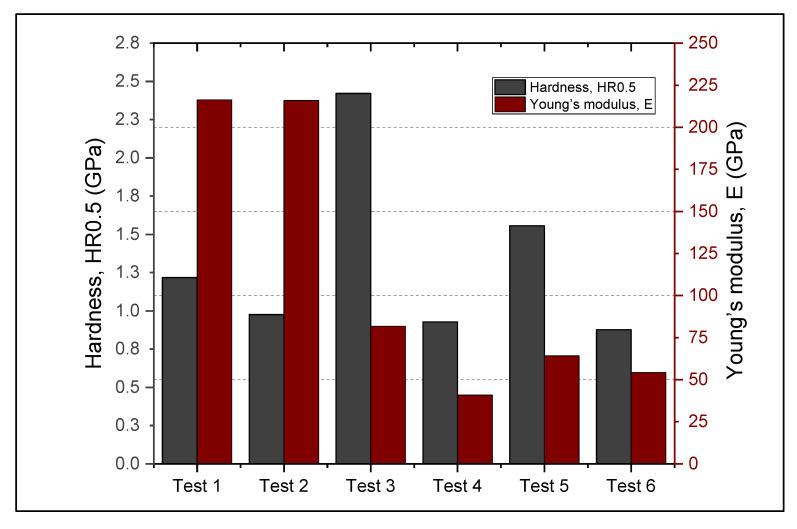
HR0.5 microhardness and Young’s modulus.

When the procedure for Rockwell microhardness HR measurements was adopted, it was considered the maximum displacement of the indenter (approximately 15 μm), far below the minimum coating thickness (over 55 μm). The adopted HR_0.5_ procedure consisted of applying a preload of 0.5 N and a continuous increase up to 5 N. The values of the microhardness and elastic modulus were computed from five similar measurements (Figure 4). The results for microhardness and Young modulus are presented in Table 2, with the confidence limits.

As can be observed from the measured mean values of the coatings, Metco 32 powder has better affinity for RUL2 substrate, with an average thickness of the coating of 126.47 µm, while Metco 72 proved better for 40Cr10 substrate (91.7 µm of coating). The worst deposition rate was obtained for Metco 72 on RUL2 substrate, the average thickness of the coating being approximately 49.74 µm. As subsequent friction tests will prove, all layers underwent slight plastic deformation and abrasion, with a final steady frictional torque evolution, the SEM analysis proving that no layer was completely removed from the base material.

The best microhardness values were obtained for Metco 32 coatings regardless of the substrate. The confidence limits presented in Table 2 show that the microhardness and Young’s modulus values were influenced by the rough and porous structure of the coating. Mrdak et al. [36] found that the microhardness of APS coatings depends on spraying distance. In our research, the spraying distance was kept constant (150 mm). At the microhardness level, each indentation point presents different roughness, porosity and chemical composition, the spread of the indentation results being of the same order as measured by Vashishtha et al. [37] for HVOF coatings of WC-12Co and WC-10Co-4Cr. The corresponding hardness values of the base materials are in good agreement with the existing materials databases [38], with near microhardness and Young modulus values obtained. However, the results of our indentation tests show a slightly better hardness of 40Cr10 steel of 1.22 GPa, while for RUL2 steel it was 0.99 GPa.

### 3.2. Surface Topography

For all the samples used in dry and lubricated tests, the arithmetic mean roughness, Ra, of each sample was measured in the circumferential (rolling) direction. The results of the profilometer measurements are presented in Table 3. For the roughness analysis, the adopted parametric values are: cutoff (Lc) 0.25 mm, cutoff (Ls) 0.008 mm, Gaussian filter, bandwidth 30:1. The lower rotating disc, made of AISI 52,100 steel, employed in friction and wear tests on the AMSLER machine, was polished each time by using sandpaper grit 320. In such a way, a similar roughness of the AISI 52,100 disc was kept constant during all the friction experiments. The obtained circumferential roughness of the lower disc was 1.0 ± 0.1 μm, and the transversal (axial) roughness was 1.2 ± 0.1 μm.

The two batches of tested rollers in dry and grease-lubricated conditions confirmed roughness of the same magnitude, proving the quality of the APS deposition technique on cylindrical rotating surfaces. It must be mentioned that the obtained surface quality was maintained during tests, with no further polishing of the coatings. The surface of the uncoated samples was obtained by turning. As the results of the friction tests will show, the roughness values are greatly influencing the friction torque and the friction coefficient in grease-lubricated conditions. The variation in the roughness values is typical to the nature of the APS deposition process, the successive passes of the deposition gun over the base material conducting to erratic splat-on-splat melted powder structures.

### 3.3. Microstructural Characterization of the Coatings by SEM Analysis

#### 3.3.1. Surface SEM Analysis

**Figure 5:**
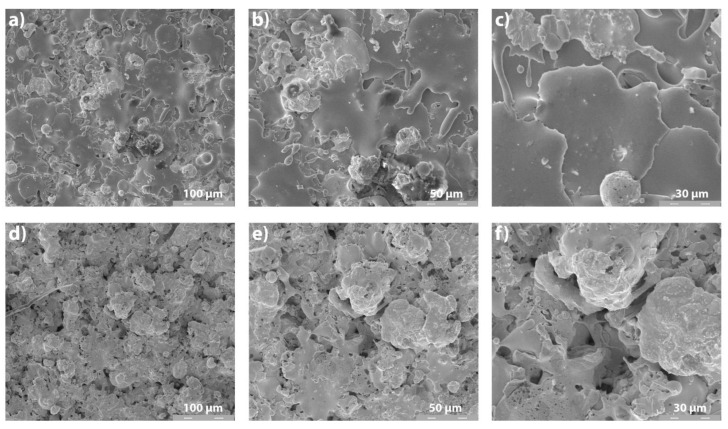
Surface SEM analysis of the coatings: (**a**) Metco 32, 500×; (**b**) Metco 32, 1000×; (**c**) Metco 32, 2000×; (**d**) Metco 72, 500×; (**e**) Metco 72, 1000×; (**f**) Metco 72, 2000×.

Surface SEM images of Metco 32 and Metco 72 coatings are presented in Figure 5. Metco 32 spheroidal particles resulted in a multi-layer splatted-like flakes structure. Correlated with the roughness measurements, one can observe that the resulting roughness of both Metco 32 and Metco 72 is in the range of 4.0 μm up to 8.0 μm, the boron and silica elements from Metco 32 assuring good melting of the powder and the same surface roughness as obtained for the smaller particles with irregular form of Metco 72 powder.

In addition, the coated surfaces possess a morphology characteristic to the APS deposition process, with splats, pores, and rare partially melted powder particles.

#### 3.3.2. Cross-Section SEM Analysis of Metco 32 and Metco 72 Coatings on 40Cr10 Substrate

**Figure 6:**
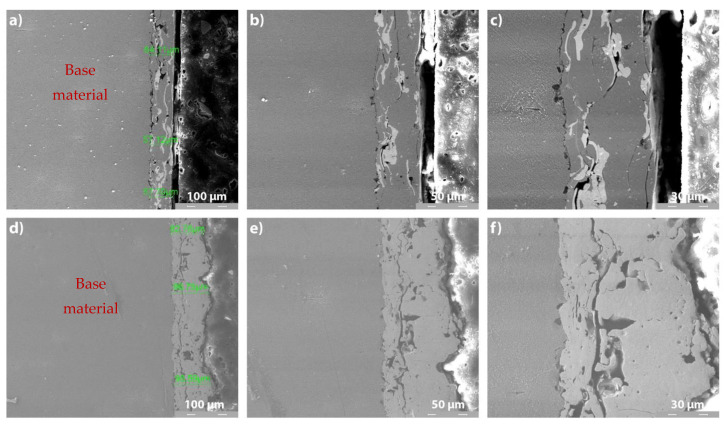
Cross-section SEM analysis of METCO 32 and Metco 72 coatings deposited on 40 Cr10 substrate: (**a**) Metco 32/40Cr10, 500×; (**b**) Metco 32/40Cr10, 1000×; (**c**) Metco 32/40Cr10, 2000×; (**d**) Metco 72/40Cr10, 500×; (**e**) Metco 72/40Cr10, 1000×; (**f**) Metco 72/40Cr10, 2000×.

**Figure 7:**
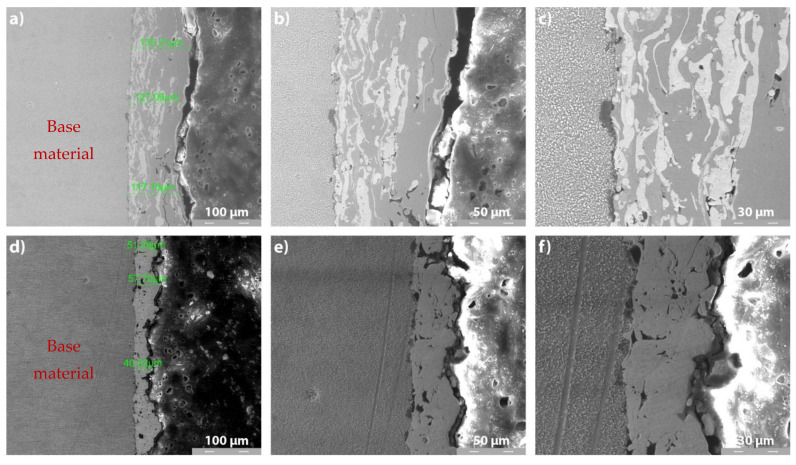
Cross-section SEM analysis of METCO 32 and Metco 72 coatings deposited on RUL2 substrate: (**a**) Metco 32/RUL2, 500×; (**b**) Metco 32/RUL2, 1000×; (**c**) Metco 32/RUL2, 2000×; (**d**) Metco 72/RUL2, 500×; (**e**) Metco 72/RUL2, 1000×; (**f**) Metco 72/RUL2, 2000×.

Figure 6 and Figure 7 present the cross-section SEM images of Metco 32 and Metco 72 powders, deposited by APS on 40Cr10 substrate (Figure 6) and RUL2 substrate (Figure 7). A relatively homogeneous structure is observed for all the coatings, with the presence of partially melted particles and the appearance of a few micro-cracks. These microcracks at the interface were generated due to internal stresses arising from non-uniform shrinkage and high particle temperature during the cooling process. These aspects are characteristic of the APS deposition process. It is obvious that the coatings adhered well to the substrate, but some micropores appeared at the substrate–coating interface. Furthermore, the average thickness of the deposited layers differs from a coating to another and from a substrate to another. The differences in layer thicknesses may result from the different sizes of the constituent particles of the powders and the different affinity of the melted particles to the substrates. Microstructure in Metco 32 coatings shows a spall-like structure evidenced by the formation of Ni-based compounds with a light-colored lamellar appearance.

The base materials have a predominantly pearlite structure, with the occurrence of primary and secondary carbides, carbides that impart hardness and wear resistance. The APS coatings showed uniform, compact layers with the presence of micropores and micro-cracks and some semi-melted particles. Regarding the microstructural analysis of APS-deposited Ni alloys coatings, Zhang et al. [39] underline that the darker color on the SEM images represents unmelted particles and pores (see Figure 6c,f and Figure 7c,f at 2000×). The unmelted particles and partially melted particles are produced due to a low rate of gas flow or high powder feed rate. The presence of close-placed spherical-shape pores into the coating has as the main source of the entrapped gases generated in the spraying process. The intra-lamellar cracks occur within the splats and are formed because of the thermal shrinking of the splats during rapid solidification.

### 3.4. EDS Analysis

**Figure 8:**
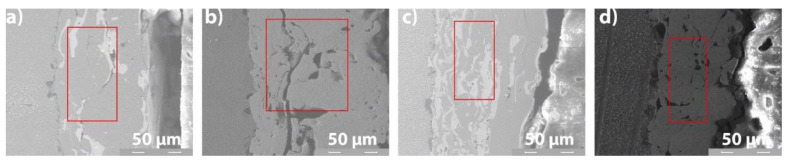
Selected cross-section middle area (2000× SEM images) of coatings: (**a**) Metco 32/40Cr10; (**b**) Metco 72/40Cr10; (**c**) Metco 32/RUL2; (**d**) Metco 72/RUL2.

**Figure 9:**
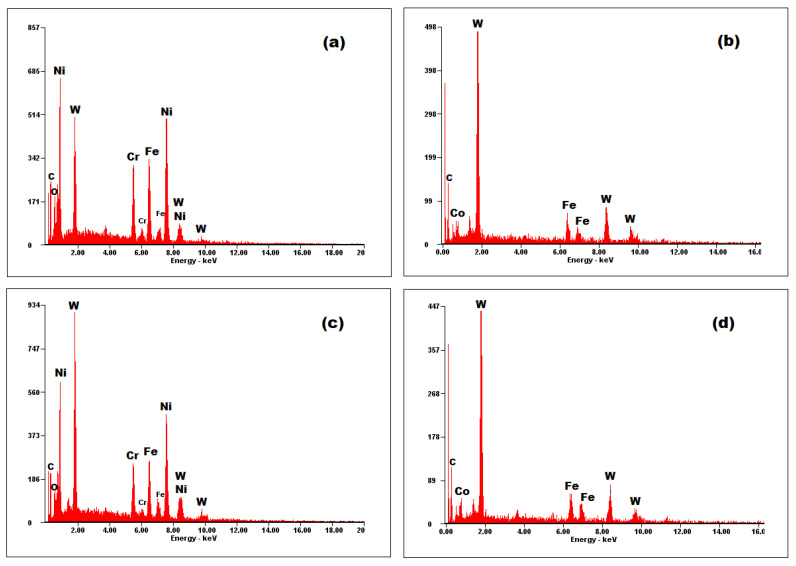
EDS spectra of coatings: (**a**) Metco 32/40Cr10; (**b**) Metco 72/40Cr10; (**c**) Metco 32/RUL2; (**d**) Metco 72/RUL2.

**Figure 10:**
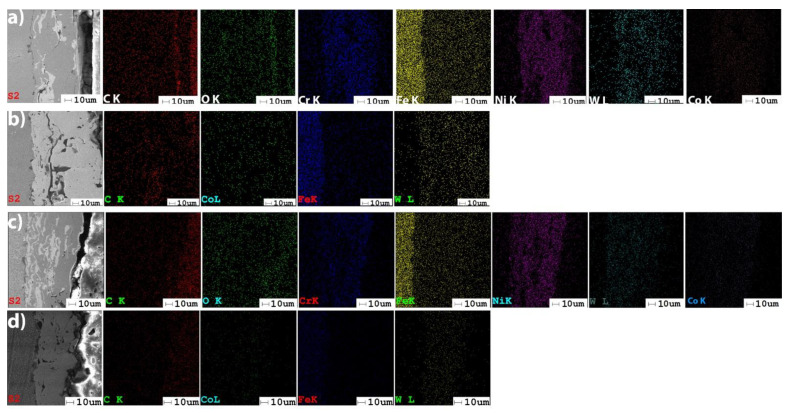
EDS maps of the distribution of elements in coatings: (**a**) Metco 32/40Cr10; (**b**) Metco 72/40Cr10; (**c**) Metco 32/RUL2; (**d**) Metco 72/RUL2.

The EDS results of the coatings provide the elemental chemical composition, in weight percent, wt.%. The measurements were obtained in the middle cross-section area of each coating, as presented in Figure 8. A comparison between the composition of the deposited powders (see Section 2.1) and the values obtained by the EDS analysis (Figure 9), shows the tendency of iron (Fe) to diffuse in coatings. Metco 32 powder already contains some percent of Fe into its soft phase (matrix). The EDS spectra of the coatings shown in Figure 9 correspond to the area marked in Figure 8. Figure 10 presents the EDS maps of the distribution of elements in coatings.

In Metco 32 powder, nickel and chromium have the role of sustaining oxidation and corrosion resistance at high temperatures, increasing the hardness of the coating. The low percent elements from the Metco 32 powder, as boron and Si, representing 4.0 wt.% from 20 wt.% of the matrix (soft phase), are not detectable by EDS. Anyway, boron has the role of lowering the melting temperature, helping the formation of hard phases, especially with Ni-Ni_3_B [40], and supplementary augmenting the hardness of the coating. During the melting process, this element formed Ni compounds. Silicon is added to increase the self-melting properties of the exposed alloy. The presence of WC–Co in Metco 72 significantly contributes to increased hardness. Powdered tungsten can create so-called “self-bonding” coatings because it belongs to the category of metals with high metallurgical or diffusion bonding capabilities. These diffusion bonds are produced locally at shallow depths of max. 0.5 μm, [41].

### 3.5. Friction and Wear Results

#### 3.5.1. Friction Results

As specified in Section 2.2.5, the friction characteristics of the Metco 32 and Metco 72 coatings, deposited on both Cardan 1 (40Cr10) and Cardan 2 (RUL2) base materials, were tested on the AMSLER tribometer in dry and grease-lubricated conditions. The mean values of the coefficients of friction (CoF) computed for three identical tests are indicated in Table 4.

**Figure 11:**
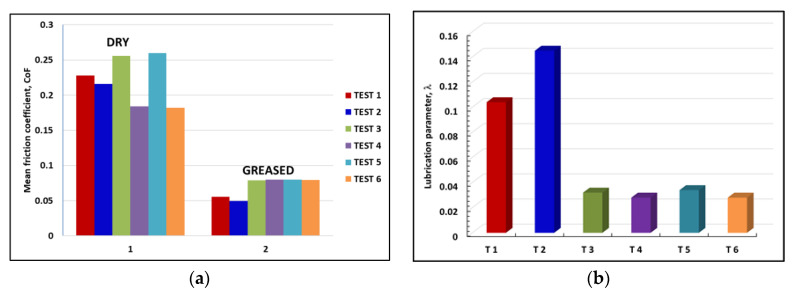
(**a**) Coefficient of friction mean values for the two batches of tested specimens, 1 and 2, and various tribological pairs in dry and grease lubrication (Test 1 to Test 6); (**b**) lubrication parameter, λ, in grease-lubricated tests.

The batches 1 and 2 of testing specimens were tested in dry and grease-lubricated conditions, respectively. The variation in the CoF for both tested batches is represented in Figure 11a.

It can be seen that even though the load was doubled from 20 to 40 N, the average CoF values decreased by an order of magnitude in the grease tests compared to the dry tests. For dry and grease-lubricated tests, the corresponding Hertz pressure value, computed according to [28], is 0.574 and 0.723 GPa, respectively. It seems that during rapid tests the lubricant (Mobilux EP2 grease) successfully separated most of the mating surfaces asperity contacts between the lower AISI52100 steel roller and the upper discs testing specimens. The better surface finishing of the uncoated specimens, Test 1 and Test 2, assured lower friction coefficients, as the lubrication regime is dictated by the λ lubrication parameter, given by Equation (1), [42]:

(1) $$\lambda =\frac{h_{min}}{\sqrt{R_{a\_lower}^{2}+R_{a\_upper}^{2}}}$$

where $h_{min}$ is the minimum lubricant film thickness, in μm, computed according to the Hamrock and Dowson formula, presented in [43], and $R_{a\_lower}$ and $R_{a\_upper}$ are the measured arithmetic mean roughness values of the lower and upper discs, respectively. Neglecting the influence of the coating micrometric layer on the overall Young modulus of the coated discs, the corresponding mean values for the λ lubrication parameter in grease lubrication conditions are indicated in Table 5, taking into account the real roughness values of the tested specimens. It can be seen that the better roughness is very important, as the composite surface roughness term, $\sqrt{R_{a\_lower}^{2}+R_{a\_upper}^{2}}$, plays a crucial role and dictates the type of the lubrication regime.

The variation in lubrication parameter, λ, in grease-lubricated tests is presented in Figure 11b. The CoF variation should follow the inverse variation in the lubrication parameter in different tests because better lubrication conditions mean lower friction. In conclusion, the higher the λ parameter, the lower the CoF values that must be obtained. The values of the lubrication parameter correspond for uncoated surfaces with better roughness to the mixed lubrication regime, that is preponderant grease separation of the mating roughness of lower and upper discs, with occasional asperity contacts. For the “as obtained” unpolished coatings, the λ parameter indicates a boundary lubrication regime, with preponderant direct contacts between the mating asperities of the rough surfaces and occasional grease separator lubricating film. From this combined theoretical and experimental interpretation of the results, the conclusion is that after the APS deposition process, the roughness of coatings should be reduced by proper polishing to ensure better lubrication and lower friction.

For the dry tests (Figure 11a), the results showed that tests 4 and 6, i.e., Metco 72/40CR10 and Metco 72/RUL2, gave lower CoF values. This can be also explained by the decreased microhardness of Metco 72-deposited coatings obtained by the APS process, which is approximately half of the microhardness for Metco 32 coatings (see Table 2).

#### 3.5.2. Wear Mechanism

As the needle roller bearings equipping the cardan spindles are always grease lubricated and sealed, only the wear SEM images of the lubricated tests are presented herein.

**Figure 12:**
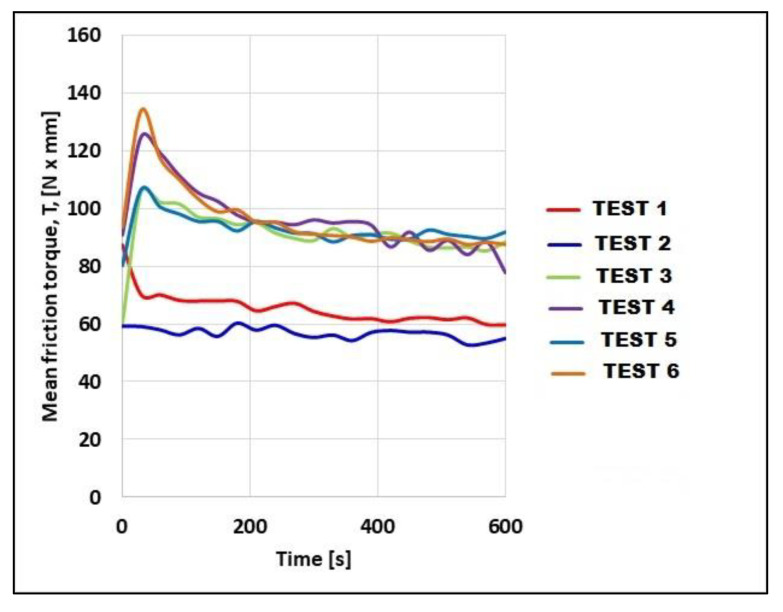
Evolution of mean friction torque in grease-lubricated tests.

**Figure 13:**
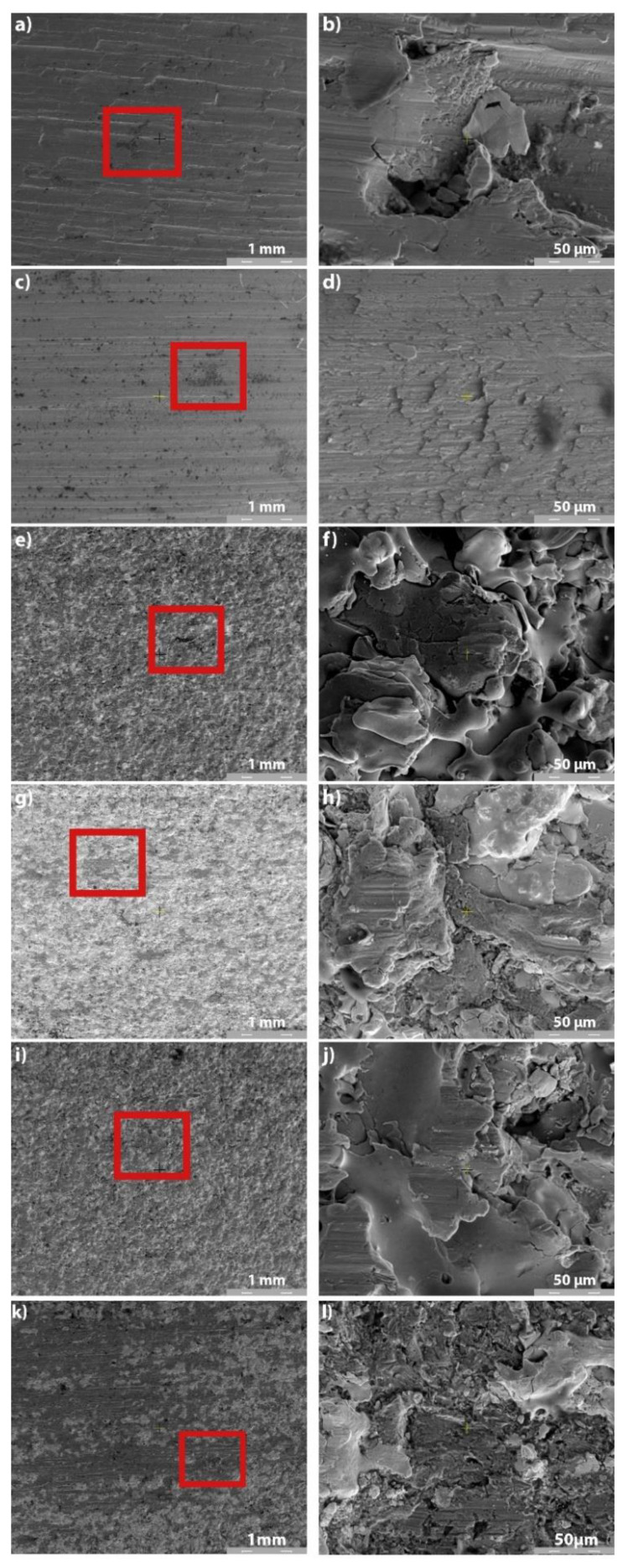
SEM images of tested samples in grease-lubricated environment: (**a**) Test 1 (100×); (**b**) Test 1 (2000×); (**c**) Test 2 (100×); (**d**) Test 2 (2000×); (**e**) Test 3 (100×); (**f**) Test 3 (2000×); (**g**) Test 4 (100 ×); (**h**) Test 4 (2000×); (**i**) Test 5 (100×); (**j**) Test 5 (2000×); (**k**) Test 6 (100×); (**l**) Test 6 (2000×).

To establish the wear modes of the uncoated and coated samples, the variation in the mean friction torque T (Figure 12) must be correlated with SEM images of the wear paths at different magnifications (Figure 13), mean roughness and hardness values of mating surfaces, and mean CoF values. In Test 1 (uncoated sample of 40Cr10), the friction was intensified at the beginning of the test (Figure 12, Test 1), indicating a running-in period accompanied by intense abrasion and plastic deformation of the roughness (Figure 13a). Figure 13b shows rare crushed abrasion particles entrapped in the surface of the testing specimen. The evolution of the friction torque in Test 2 (uncoated RUL2 specimen) was constant and the sporadic damages look like abrasion wear (Figure 13c), and small wear particles attached to the surface of the tested sample (Figure 13d). The friction torque in Test 3 (Metco 32/40Cr10) and Test 5 (Metco 32/RUL2) is similar (Figure 12), and also the wear mode is the same. At a lower magnification of 100×, the ceramic coating of Metco 32 looks intact (Figure 13e,i), excepting a few regions with deformed splatted particles (Figure 13f,j). From the SEM images, it is clear that the Metco 32 coating wears by plastic deformation of the top roughness and abrasion. The microtopography of the two Metco 32 coatings is almost the same with near roughness values, but the hardness of Metco 32/40Cr10 (Test 3) is higher than that of Metco 32/RUL2 (Test 5), from here an intensified wear resulted for the sample from Test 5 (Figure 13j). For the Metco 72 coatings, more abrasive damage can be observed for Test 6 (Metco 72/RUL2), Figure 13l, its hardness a little lower than that of the coating from Test 4 (Metco 72/40Cr10), Figure 13h. For this reason, even if the roughness of the two coatings is almost the same, the friction torque is a little intensified at the beginning of Test 6. The running-in period of all the coated samples is the same, approximately 200 s (Figure 12), but for the coating of Metco 72, the friction was more intense at the beginning of the tests.

It should be noted that all coatings remained on the substrate, with only superficial plastic deformation of roughness and mild abrasive wear. In the dry friction tests, the lowest values of the mean friction coefficients were obtained for the Metco 72 coating, but in the lubricated tests, the CoF values of both coatings were almost the same. In any case, wear tests and SEM images proved that Metco 32 performs well with grease lubrication, with mild abrasive wear and no significant damage to the coating surface observed. Considering the hardness of the coatings, which will dictate the wear resistance in the long-lasting functioning of cardan joints, a good coating could be Metco 32 on 40Cr10 substrate, with the highest micro-hardness and low CoF in grease lubrication. Anyway, for the dry running of cardan joint bearings, the best coating is Metco 72 on 40Cr10, with the lowest CoF.

**Figure 14:**
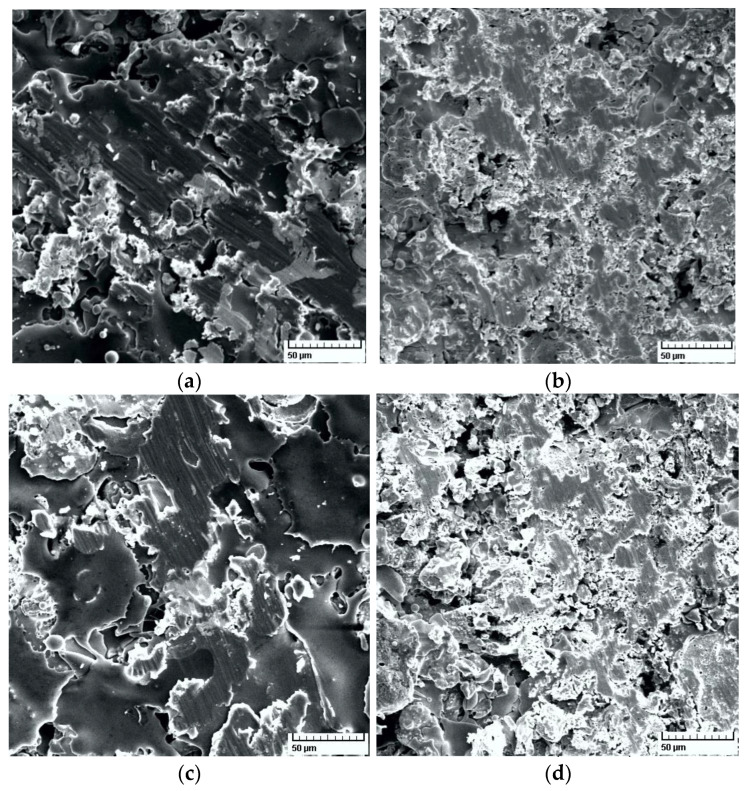
SEM images of scratch tests (500×): (**a**) Metco 32/40Cr10; (**b**) Metco 72/40Cr10; (**c**) Metco 32/RUL2; and (**d**) Metco 72/RUL2.

The results of the AMSLER tribometer friction tests should be correlated with the results of the scratch tests performed on the four combinations of coatings and substrates shown in Table 6. Additionally, some significant SEM images of the scratch tests are presented in Figure 14.

It can be seen that the average CoF in the scratch tests is higher for the RUL2 substrate regardless of the applied coating, suggesting better adhesion of the coating to the substrate. The best adhesion was found for the Metco 72 layer on the RUL 2 substrate, interestingly with the lowest thickness of all layers (approximately 49 µm). In terms of the wear modes manifested in the scratch tests, the results correlate well with the results of the friction tests (Figure 14). The worn surfaces of the coatings subjected to scratch tests show cut particles. The lower hardness of the Metco 72 coating resulted in an extended worn surface, with the largest worn surface for Metco 72/40Cr10 (Figure 14b). The smallest area with deformed particles was obtained for Metco 32/RUL2 (Figure 14c), the result consistent with those obtained from friction tests.

These new results, correlated with the previous ones obtained from the friction torque evolution during AMSLER tests (Figure 12), recommend Metco 32/RUL 2 (test 5) as the best coating and substrate solution, the friction coefficient evolution during the running-in period being more favorable than that obtained for the Metco 72/RUL 2 coating. In addition, it was found that the microhardness of the Metco 32 coating was higher than that of Metco 72 (see Table 2). There was good correlation between the results of microhardness, friction and scratch tests, and SEM analysis.

**Figure 15:**
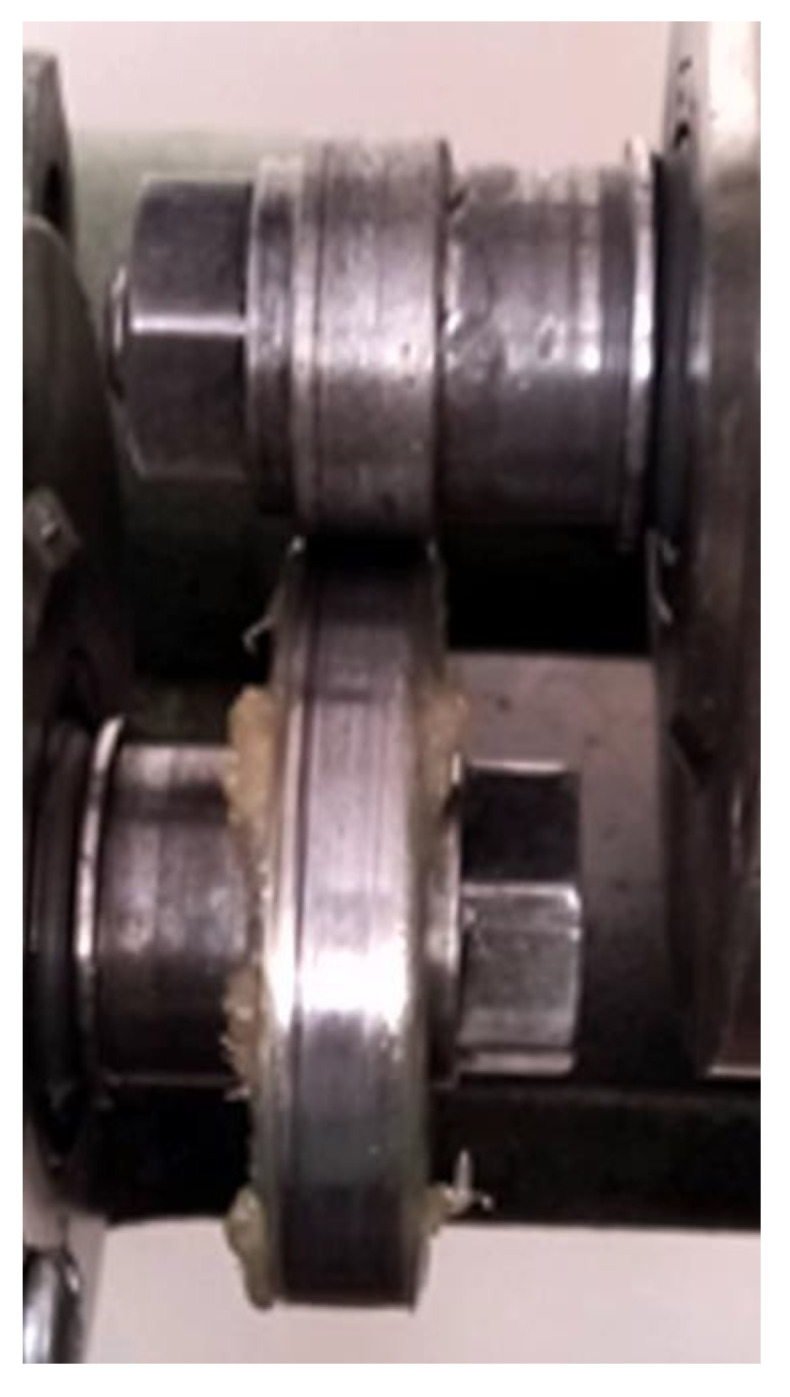
Grease expulsion from the contact.

However, an unexpected phenomenon of grease expulsion from the contact occurred in all lubricated tests, the grease was trapped on the lower roller of AISI521000, due to its higher tangential speed, and was removed from the contact towards the edge of the lower roller (Figure 15).

Figure 15 shows that the larger-diameter disc entrains most of the grease and expels it outwards, the contact being lubricated at the end of the tests with a tiny amount of grease. Correlating this information with friction and wear results for both dry and lubricated tests, it results that the coatings ensure better protection to wear than the bare steel 40Cr10 and RUL2. In any case, the expulsion of grease from the contact indicates that, in actual application, the grease is attracted to the spindle of the cardan cross and pushed against the seals, leaving the needle rollers poorly lubricated. This fact explains why the spindles of the cardan crosses fail by abrasive wear and pitting. In needle bearing cardan joint operation, grease will tend to be expelled towards the seals. Any cracking or deterioration of the shaft and bearing seals, for example, due to salts deposited on the road surface in winter, will lead to a change to dry friction.

From the graph of the variation in the average friction torque (Figure 12) under grease lubrication conditions, it can be seen that the roughness of the sample dictates the magnitude of the friction torque. One solution to reduce friction and reduce wear is to polish Metco coatings to a lower roughness, and future tests will focus on this as well.

## 4. Conclusions

The Metco 32 and Metco 72 coatings on 40Cr10 and RUL 2 substrates were prepared by APS. The indentation tests showed that the microhardness of the Metco 32 coating is higher than that of Metco 72. The surface and cross-section SEM analysis of the coatings revealed their splatted morphology with some partially melted particles, micro-cracks and micropores, specific to the APS process. The deposition parameters in the APS process were kept constant and the number of passes of the deposition gun over the samples was the same; coatings with uniform thickness were obtained, their thickness depending on the substrate and coating type.

Two batches of six samples each, obtained by combining the substrates and the coatings and including also the uncoated specimens, were tested on AMSLER machine in friction and wear tests at a constant speed and constant load for both dry and grease-lubricated conditions, the obtained results correlating with the results of the scratch tests. Following the results of the friction tests and SEM analysis, it was noticed that all coatings remained on the substrate with only superficial plastic roughness deformation and slight abrasive wear. In the dry friction tests, the lowest mean CoF values were obtained for the Metco 72 coating, but in the lubricated tests, the CoF values of both coatings were almost identical irrespective of the substrate.

The average CoF in scratch tests is higher for the RUL2 substrate regardless of the applied coating, suggesting better adhesion of the coating to the substrate. Even if the best adhesion was found for the Metco 72 coating on RUL 2 substrate (test 6), the better hardness of the Metco 32 coatings, the uniform evolution of the CoF during running-in period of the friction tests and the results of the SEM analysis on worn surfaces are better for Metco 32/RUL2 (test 5), recommending Metco 32/RUL2 as the best coating and substrate solution.

In lubricated tests, it was observed that most of the grease is transferred from the smaller-diameter disc to the larger-diameter disc due to the different peripheral velocities, with the lower disc with higher peripheral velocity taking up the grease. In addition, the grease is expelled to the outside of the lower disc, the contact remaining with a minute amount of grease. It is possible that over a longer period, the more porous coatings may form micro-lubricant reservoirs, and supplementary future long-term testing is needed to elucidate this.

## Figures and Tables

**Table 1:** Materials and tested samples.

Test Number	Lower Disk	Upper Disk	
		Substrate	Coating
TEST 1	AISI52100	40Cr10	without
TEST 2	AISI52100	RUL2	without
TEST 3	AISI52100	40Cr10	Metco 32
TEST 4	AISI52100	40Cr10	Metco 72
TEST 5	AISI52100	RUL2	Metco 32
TEST 6	AISI52100	RUL2	Metco 72

**Table 2:** Young’s elasticity modulus, E, and Rockwell microhardness HR0.5.

Parameter	Metco 32/40Cr10	Metco 72/40Cr10	Metco 32/RUL2	Metco 72/RUL2	40Cr10	RUL2
Microhardness, HR_0.5_ [GPa] (and confidence limits)	2.42 (−0.37; +0.48)	0.93 (±0.08)	1.56 (−0.28; +0.31)	0.88 (−0.15; +0.3)	1.22 (±0.3)	0.98 (±0.2)
Young modulus, E [GPa] (and confidence limits)	81.8 (−7.51; +11.43)	40.88 (±3.25)	64.21 (−8.68; +4.80)	54.34 (−6.93; +7.18)	216.33 (±5.11)	215.93 (±1.04)
Coating thickness, hc [μm] (and confidence limits)	59.64 (−4.47; +2.52)	91.70 (−5.05; +8.94)	126.47 (−8.74; +9.32)	49.74 (−7.96; +9.52)	0 -	0 -

**Table 3:** Roughness for the batches of tested samples (upper discs), R_a_.

Sample	Testing Conditions	
	Dry	Grease Lubricated
	Roughness Ra, [μm]	Roughness Ra, [μm]
40Cr10 (no coating)	1.96 ± 0.11	1.04 ± 0.07
RUL 2 (no coating)	0.37 ± 0.04	0.28 ± 0.03
Metco 32/40Cr10	7.54 ± 0.29	4.61 ± 0.14
Metco 72/40Cr10	5.97 ± 0.26	5.22 ± 0.21
Metco 32/RUL2	5.55 ± 0.24	4.31 ± 0.17
Metco 72/RUL2	4.44 ± 0.19	5.19 ± 0.24

**Table 4:** Average coefficient of friction for dry and grease-lubricated tests.

Test No. ^1^	Test 1	Test 2	Test 3	Test 4	Test 5	Test 6
Dry CoF (and confidence limits)	0.228 (±0.021)	0.216 (±0.019)	0.256 (±0.027)	0.184 (±0.016)	0.260 (±0.03)	0.182 (±0.019)
Greased CoF (and confidence limits)	0.0556 (±0.0041)	0.0496 (±0.0037)	0.079 (±0.0057)	0.0801 (±0.0062)	0.0797 (±0.0059)	0.0795 (±0.0054)

^1^ Test number corresponds to Table 1.

**Table 5:** The lubrication parameter, λ, for grease-lubricated tests.

Test No. ^1^	Test 1	Test 2	Test 3	Test 4	Test 5	Test 6
λ parameter	0.104	0.145	0.032	0.028	0.034	0.028
Lubrication regime	Mixed		Boundary			

^1^ Test number corresponds to Table 1.

**Table 6:** Average coefficient of friction during the scratch tests.

Coating/ Substrate	Metco 32/ 40Cr10	Metco 72/ 40Cr10	Metco 32/ RUL2	Metco 72/ RUL2
Average dry CoF Standard deviation	0.481 0.190	0.550 0.205	0.604 0.240	0.626 0.225
